# Genetic Divergence and Antibody Expression Influence the N‐Glycomes of CHO‐K1 and CHO‐S Cells

**DOI:** 10.1002/bit.70045

**Published:** 2025-08-16

**Authors:** Roberto Donini, Pat Blundell, Richard J. Pleass, Dongli Lu, Anne Dell, Cleo Kontoravdi, Stuart M. Haslam

**Affiliations:** ^1^ Department of Life Sciences Imperial College London London UK; ^2^ Department of Tropical Disease Biology Liverpool School of Tropical Medicine Liverpool UK; ^3^ Department of Chemical Engineering Imperial College London London UK

**Keywords:** antibody glycoengineering, biopharmaceuticals, Chinese hamster ovary (CHO) cells, MALDI‐TOF, mass spectrometry, N‐glycosylation

## Abstract

While Chinese hamster ovary (CHO) cells continue to be the workhorse of recombinant therapeutic protein production, decades of genetic divergence in the industrially relevant CHO‐K1 and CHO‐S cell lines, are likely to have resulted in differing glycosylation capabilities. Glycosylation can influence the efficacy, serum half‐life, and safety of biologics, and as a critical quality attribute of glycoprotein biopharmaceuticals, it is essential to better understand how major CHO cell manufacturing platforms diverge. We used matrix‐assisted laser desorption ionization‐time of flight mass spectrometry to perform N‐glycomic analyses comparing CHO‐K1 cells, CHO‐S cells and antibody‐producing daughter cell lines. The results reveal that genetic divergence in these industrially relevant cell lines, as well as the burden of antibody production, lead to significant differences in antennal branching and terminal elaboration in the cellular N‐glycome. More specifically, CHO‐K1 cells produce larger and more complex N‐glycans with higher levels of sialylation than CHO‐S cells, and antibody production was associated with increased antennal branching. Additionally, these findings were also reflected in the N‐glycomic profiles of IgG1‐Fc constructs produced in either CHO‐K1 and CHO‐S cells.

## 1 Introduction

Chinese hamster ovary (CHO) cells are currently the most important manufacturing platform for the multibillion USD biopharmaceutical industry. From 2018 to 2022, nearly 70% of approved biopharmaceuticals, and 85% of novel macromolecules (i.e., excluding biosimilars), were produced in CHO cells (G. Walsh and E. Walsh 2022). An even larger proportion of monoclonal antibodies (mAbs), 89%, were produced in these hosts (G. Walsh and E. Walsh 2022). In the decades since the first biopharmaceuticals were clinically approved, CHO cells came to dominate the manufacturing landscape partly because of human‐compatible glycosylation profiles and high production titers (10–15 g∙L^−1^) (Puetz and Wurm 2019). Despite the advantages of such a platform, glycosylation is not a template‐driven process and remains a heterogenous affair even in CHO cells. Enzyme competition for substrates in the cellular glycosylation reaction networks generates a mixture of glycan structures on glycoprotein therapeutics. N‐ and O‐glycosylation both directly and indirectly modulate biopharmaceutical efficacy, serum half‐life, and immunogenic responses, which is why increased control over CHO cell glycosylation and in vivo glycoengineering are useful strategies that require a deep understanding of CHO cell glycosylation capabilities. While glycosylation is not present in all recombinant therapeutic proteins and can be engineered out when functionally redundant, it remains a critical quality attribute of glycoprotein therapeutics under the FDA's “Quality by Design” paradigm. In fact, reproducible glycosylation profiles are essential for the development of biosimilars that accurately mimic originator products. Furthermore, glycoengineering can serve as a foundation for diverse strategies aimed at enhancing the quality of biopharmaceuticals in the development of biobetters.

A previous mass spectrometry (MS) glycomic analysis of N‐ and O‐glycans from CHO‐Pro^−^5 cells and a panel of glycosylation mutants forms the basis of our current understanding of the CHO cell glycosylation machinery (North et al. 2010). The data reported only simple mono‐ or disialylated core‐1 O‐glycan structures (+/−NeuAca2,3Galβ1,3‐(+/−NeuAca2,6)GalNAcα‐Ser/Thr) were produced by CHO cells, however, the N‐glycomic profiles revealed spectra with high mannose (Man) N‐glycans together with a wide range of complex N‐glycans. In CHO cells, the complex N‐glycome includes a mixture of bi‐, tri‐, and tetra‐antennary N‐glycans that can form large structures with highly extended antennae including up to 26 lactosamine (LacNAc; Gal‐GlcNAc) units. The antennae can also be capped by *N*‐acetylneuraminic acid (NeuAc sialic acid) and fucose (Fuc) was found to be present only in the chitobiose core. While these experiments generated a wealth of glycomic data for CHO cells, the Pro^−^5 cells in this study are not the dominant cell line used in industry for the manufacturing of biopharmaceutical products. In fact, CHO‐K1 and CHO‐S cells are some of the most commonly used cell lines for industrial bioprocesses in the pharmaceutical industry (F. Wurm and M. Wurm 2017). It is not possible to exactly determine the precise pedigree of CHO cell lines in use today, however, a review by Wurm and Wurm traces the origins of the CHO‐K1 cell line to the late 1960s, whereas the CHO‐S cell line emerged in the 1970s (F. Wurm and M. Wurm 2017). The initially immortalized CHO cells, and all the CHO cell lines that have since emerged, were released from the constraints imposed by diploidy control, and subsequently evolved with higher rates of DNA mutations and changes in gene expression profiles (F. Wurm and M. Wurm 2017). Inherent genetic instability in CHO cells and a lack of “true” clonality is not necessarily an issue for the reliable production of high‐quality biopharmaceuticals. However, it is likely that over decades of culturing—often in a variety of conditions that alter selective pressures—and many cell division events, the genetic divergence between, and indeed, within CHO “cell lines” could be of significance. While clonal selection is a standard process in industry that includes glycosylation as a selection criterion, genetic divergence may increase with each cell division event, potentially leading to heterogenous glycosylation capabilities and loss of “clonality.” We posited that such genetic divergence might be reflected in the expression levels of glycosylation genes and consequently affect the N‐glycosylation profiles of different CHO cell lines and the recombinant glycoprotein therapeutics produced in these cells. A genomic comparison of the CHO‐K1, CHO‐S, and CHO‐DG44 cell lineages previously highlighted such genetic differences and suggested that further biochemical characterization of glycomic phenotypes was necessary (Lewis et al. 2013).

Herein, we present mass spectrometric N‐glycomic data that highlight differences in cellular N‐glycosylation in two industrially important CHO cell lineages: CHO‐K1 and CHO‐S. Our focus on N‐glycan profiles stems from the fact that most glycosylation in recombinant biopharmaceuticals, such as IgG mAbs, is N‐linked. Additionally, CHO cell O‐glycosylation is limited to simple core‐1 structures with or without sialic acid capping and is less informative about the overall glycosylation machinery of CHO cells than N‐glycans. Furthermore, we analyzed the N‐glycome of IgG mAb‐producing cells derived from CHO‐K1 and CHO‐S cells to determine whether the production and secretion of mAbs can itself influence the glycosylation machinery of CHO cells. The impact of increased metabolic pressure from the expression and secretion of recombinant mAbs on CHO cell N‐glycosylation is not sufficiently understood. In fact, as the cell redirects limited resources toward antibody production away from biomass production, and the flux in the secretory pathway increases, the expression of glycosyltransferases and glycosidases, and the Golgi residence time of recombinant glycoproteins may be affected (Fan et al. 2015; Jimenez del Val et al. 2016). Finally, we analyzed the N‐glycomic profiles of a panel of antibody‐Fc constructs with novel glycosylation sites, produced in CHO‐S cells, and compared these to previously published data for the same constructs produced in CHO‐K1 cells. To our knowledge, this is the first study investigating the cellular glycome of industrially relevant CHO cells and the impact of recombinant product expression and secretion on the glycosylation machinery of CHO cells.

## 2 Materials and Methods

### 2.1 CHO Cell Culture

All cells were grown in CD‐CHO medium (Gibco, ThermoFisher Scientific, USA) with the relevant supplements added as shown in Table 1. Solutions were prewarmed to 37°C and cells were incubated at 37°C with 5% CO_2_ and orbital shaking (16 mm orbit; SSL1 orbital shaker, Stuart) (see Table 1). All centrifugations of CHO cells were performed at 800 rpm (120*g*; TX‐400 rotor, ThermoFisher Scientific, USA) for 5 min. During cell revival, a 1 mL cell vial stored in liquid nitrogen (LN_2_) was thawed and resuspended in 9 mL prewarmed medium. Cell density and culture viability were measured using a haemocytometer and trypan blue exclusion, and an NC‐3000 NucleoCounter with solution 18 and A8 NC‐slides according to manufacturer's instructions (Chemometec, Denmark). Cells were then seeded at the appropriate density (see Table 1) in sterile vented flasks with 50 mL of total culture volume and split every 3 or 4 days. Each cell line was cultured in biological triplicates and cells were harvested after three passages post‐revival.

**Table 1:** Cell line‐specific incubator growth conditions.

Cell line	Shaking (rpm)	Seeding density (cells·mL^−1^)	Supplements (final concentration, catalog#, manufacturer)
CHO‐K1	125	2 × 10^5^	l‐Glutamine (6 mM, #25030081 Gibco)
CHO‐S	130	2 × 10^5^	l‐Glutamine (8 mM, #25030081 Gibco) HT supplement (10 mL·L^−1^, 100× dilution, #11067030, Gibco)
CHO‐K1_mAb_	150	3 × 10^5^	l‐Methionine sulfoximine (50 μM, #GSS‐1015, Sigma Aldrich)
CHO‐S_mAb_	140	2 × 10^5^	l‐Glutamine (8 mM, #25030081, Gibco) Hygromycin B (500 μg·L^−1^, #10687010, Gibco)

### 2.2 IgG1‐Fc Constructs: Expression and Purification

The construction, expression, and purification of the CHO‐K1 panel of Fc‐fragments have been described in detail (Blundell et al. 2019, 2020), while the same panel of identical constructs manufactured in CHO‐S cells is described in Baksmeier et al. (2021).

### 2.3 N‐Glycomics

The N‐glycomic analysis is based on a previous protocol (North et al. 2010).

#### 2.3.1 Cell Harvest and Glycoprotein Extraction

On Day 4 of the third subculture, 1 × 10^7^ cells were harvested by centrifugation at 800 rpm (120*g*; TX‐400 rotor, Thermo) for 5 min. Cell pellets were washed in phosphate‐buffered saline and pelleted again prior to storage at −80°C. The cells were then sonicated in lysis buffer (25 mM Tris, 150 mM NaCl, 5 mM EDTA, and 1% CHAPS, pH 7.4) on ice—4 × 10 s in continuous mode at 40 amps (CV188 sonicator, Vibra‐Cell‐Sonics) with 1 min on ice in between each sonication step. The samples were then dialyzed against 5 × 4.5 L of 50 mM ammonium bicarbonate (pH 8.5) at 4°C for 48 h and then lyophilized. Extracted glycoproteins were then reduced with 1 mL of 2 mg/mL dithiothreitol in 0.6 M Tris buffer (pH 8.5) at 50°C for 60 min. Carboxymethylation was then carried out via the addition of 1 mL of 12 mg/mL iodoacetic acid in 0.6 M Tris buffer (pH 8.5), where samples were incubated at room temperature, in the dark, for 90 min. Samples were then dialyzed once more as described above, and lyophilized after 48 h.

#### 2.3.2 Trypsin Digestion

The reduced and carboxymethylated glycoproteins were resuspended in 700 µL of 50 mM ammonium bicarbonate and tryptically digested with the addition of 50 µL of 10 mg/mL porcine pancreas trypsin (EC 3.4.21.4; Sigma‐Aldrich) in 50 mM ammonium bicarbonate buffer (pH 8.4) at 37°C for 24 h. The reaction was terminated by adding 3 drops of pure acetic acid, and the glycopeptide products were then purified using Oasis C_18_ HLB Plus 60 µm cartridges (Waters, USA) as described previously (North et al. 2010).

#### 2.3.3 N‐Glycan Release From Glycopeptides With Peptide *N*‐Glycosidase F (PNGase F)

Glycopeptides were resuspended in 200 µL of 50 mM ammonium bicarbonate (pH 8.4) and 5 µL of recombinant PNGase F (EC 3.5.1.52, Roche, Switzerland) in glycerol was added to each sample. The reaction was carried out at 37°C for 24 h—with the addition of a supplementary 5 µL aliquot of PNGase F after the first 8 h—and then lyophilized. N‐glycans were then resuspended in 200 µL of 5% acetic acid (v/v) and separated from peptides and O‐glycopeptides by reverse phase chromatography using Sep‐Pak Classic C_18_ cartridges (Waters, USA) as described previously (North et al. 2010). N‐glycans were eluted in 5 mL of 5% acetic acid (v/v). At this stage, 1/3 of the purified N‐glycans were aliquoted for enzymatic digestion prior to derivatization, while the remaining 2/3 were derivatized directly following lyophilisation. After the aliquots were taken from the 5 mL elution, volumes were reduced on a SpeedVac, and samples were then lyophilized.

#### 2.3.4 Enzymatic Digestion of Released N‐Glycans

The 1/3 aliquots of purified N‐glycans were resuspended in 400 µL of 50 mM sodium acetate (pH 5.5) and 8 µL of endo‐β‐galactosidase (EC 3.2.1.103, R&D Systems, Biotechne, USA) was added to each sample, which were then incubated at 37°C for 24 h, and then lyophilized. Digested N‐glycans were resuspended in 200 µL of 5% acetic acid (v/v) and purified once more by reverse phase chromatography using Sep‐Pak Classic C_18_ cartridges (Waters, USA) with the same protocol as the post‐PNGase F chromatographic step. The samples were then lyophilized and derivatized.

#### 2.3.5 Derivatization of N‐Glycans

N‐glycans were permethylated with the sodium hydroxide procedure. First, 5 pellets of sodium hydroxide (per sample) were ground in a dry glass mortar with 3 mL of anhydrous dimethylsulfoxide. In total, 1 mL of the resulting slurry was then added to each sample tube together with 0.5 mL of methyl iodide and mixed vigorously with an automatic shaker for 15–45 min (until the slurry becomes a white paste that no longer mixes). The reaction was quenched by the drop‐wise addition of water with constant agitation, after which 1 mL of chloroform was added to extract the permethylated N‐glycans. The mixture was made up to ~5 mL with ultra‐pure water and vortexed vigorously for 30 s to wash out hydrophilic contaminants. The hydrophobic and hydrophilic layers were separated rapidly by centrifugation, and the aqueous layer was then discarded. A further four washes were performed by adding 5 mL of ultra‐pure water, vortexing for 30 s, and discarding the aqueous layer after centrifugation. The chloroform layer was then dried under a gentle stream of nitrogen gas.

#### 2.3.6 Purification of Permethylated N‐Glycans

Permethylated N‐glycans were further purified from hydrophilic contaminants by reverse phase chromatography using Sep‐Pak Classic C_18_ cartridges (Waters, USA). The cartridges were conditioned by eluting successively 5 mL methanol, 5 mL ultra‐pure water, 5 mL acetonitrile (MeCN), and reequilibrated with 3 × 5 mL ultra‐pure water. The dry samples from the chloroform extraction were redissolved in 100 µL methanol, after which 100 µL of ultra‐pure water was added to each sample prior to loading onto the cartridge. Once the sample was loaded, a 5 mL wash with ultra‐pure water was eluted and then 3 mL of each 15%, 35%, 50%, and 75% MeCN (v/v) were eluted and collected. All fraction volumes were reduced on a SpeedVac and samples were then lyophilized.

#### 2.3.7 MALDI‐TOF MS and MALDI‐TOF/TOF MS/MS Analysis

The lyophilized permethylated N‐glycans were redissolved in 10 µL methanol and a 1 µL aliquot from each fraction was mixed with 1 µL of 3,4‐diamino‐benzophenone (DABP) matrix solution (10 mg/mL, in 75% MeCN, v/v). 2 × 0.5 µL of the sample/matrix mixture were then spotted onto a clean matrix‐assisted laser desorption ionization (MALDI) plate and dried under vacuum. The spotted plate was then loaded into a 4800 MALDI‐TOF/TOF mass spectrometer (Applied Biosystems, USA) for MS and MS/MS glycomic analyses.

MALDI‐MS data were collected in the positive ion mode, with a mass range of m/z 500–9000. Four spectra (1000 shots each) were collected for each sample/spot with a laser intensity of 4000 and 20 kV accelerating voltage. MS/MS analyses were performed on the same instrument, however, using the positive reflectron mode, with argon gas in the collision cell (at 10^−4 ^mbar pressure) to induce collisionally activated decomposition. For these experiments, the potential difference between the source acceleration voltage and the collision cell was set to 1 kV. All structural assignments of N‐glycans were based on molecular ion composition, previous knowledge of biosynthetic pathways, and MS/MS fragmentation patterns. Data Explorer (Applied Biosystems, USA) and GlycoWorkBench were used for the analysis of MALDI‐MS and MALDI‐MS/MS spectra (Ceroni et al. 2008). Quantitative information of N‐glycans was derived from ion counts which do not consider ionization efficiencies and should therefore not be considered absolute values. The relative abundances of galactose and NeuAc sialic acid in each sample were calculated by summing the relative intensities of galactose‐, or sialic acid‐containing N‐glycans and dividing by the sum of relative intensities for all assigned N‐glycans and finally expressed as a percentage (the number of Gal or NeuAc residues in each structure were not considered in this analysis) (see Figures 3 and 8). The same method was used to analyze the relative abundance of core‐fucosylation, and oligomannose/complex N‐glycans. This data omit monoisotopic peaks corresponding to putative very low abundance afucosylated structures that might be present in isotopic clusters (see Figures S5 and S6).

## 3 Results

Samples for N‐glycomic analyses were prepared from CHO‐K1, CHO‐S, CHO‐K1_mAb_, and CHO‐S_mAb_ cell cultures grown in suspension that were pelleted, washed, and sonicated. Glycoproteins in the cell lysates were then reduced and carboxymethylated, and tryptically digested. The N‐glycans were subsequently released from tryptic glycopeptides by PNGase F digestion, purified, and permethylated to increase the sensitivity of the MS analysis. Endo‐β‐galactosidase enzymatic digests were also performed on purified N‐glycans prior to permethylation to enhance the structural characterization of cellular N‐glycans from CHO cells. This enzyme cleaves the internal β1,4‐Gal linkage of antennal LacNAc's, and the resulting MS profiles offer additional insights into complex N‐glycan branching and terminal elaboration patterns. Finally, the N‐glycans from purified IgG1‐Fc constructs produced in CHO‐S cells were also analyzed using MS and compared to previously published data for the same constructs produced in CHO‐K1 cells (Blundell et al. 2019, 2020).

We used MALDI‐MS to generate singly charged sodiated molecular ions ([M + Na]^+^). The MS spectra generated were then structurally annotated by mass fingerprinting and used to perform a semi‐quantitative analysis of relative intensities of different N‐glycan structures. Our analysis was primarily focused on comparing the relative abundances of oligomannose N‐glycans and complex N‐glycans with variable levels of fucosylation, galactosylation, and sialylation and different branching patterns. Furthermore, certain molecular ions detected in the MS spectra were analysed by MS/MS. Through collisionally activated dissociation, MS/MS generates sequence‐informative fragment ions, which help to identify or confirm antennal structures, branching patterns, and linkage positions.

### 3.1 Overview of CHO‐K1, CHO‐S, CHO‐K1_mAb_, CHO‐S_mAb_ Cell N‐Glycomes

**Figure 1:**
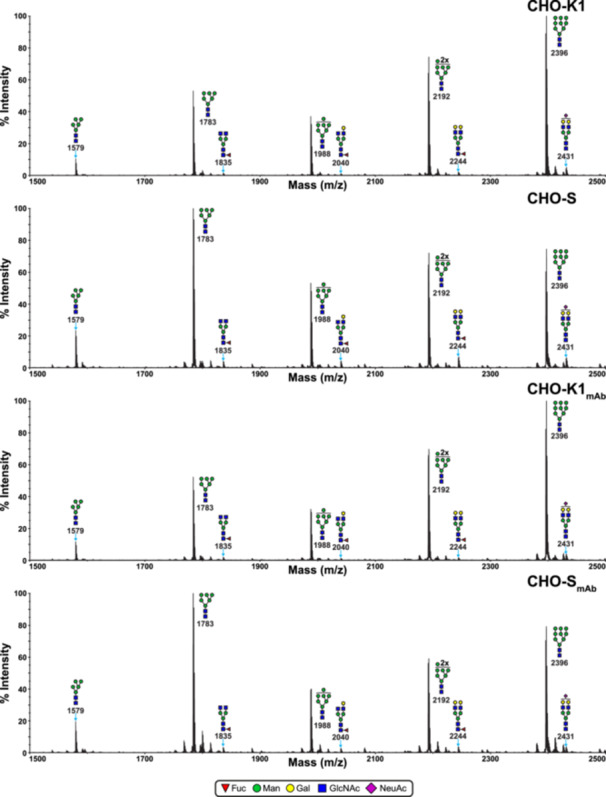
MALDI‐TOF MS profiles of low m/z range permethylated N‐linked glycans derived from CHO‐K1, CHO‐S, CHO‐K1_mAb_, and CHO‐S_mAb_ cells highlight changes in the abundance of oligomannose N‐glycans (m/z 1500–2500). All molecular ions represent the singly charged and sodiated form [M + Na]^+^. Data were obtained from the 50% acetonitrile (MeCN) fraction from a C_18_ Sep‐Pak. Structures shown with brackets have not had their antennal location unequivocally defined. Some N‐glycan structures are not annotated in these spectra in the interest of clarity—fully annotated spectra for these samples are shown in Figures S1–S4.

**Figure 2:**
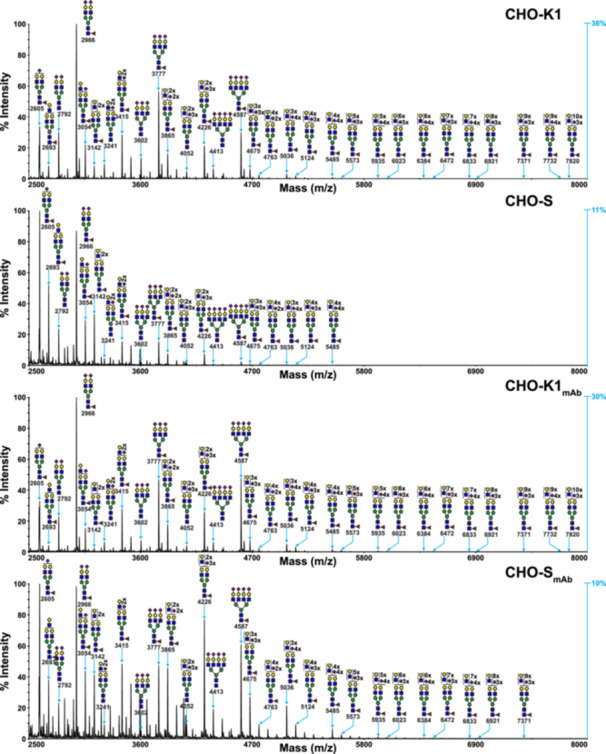
MALDI‐TOF MS profiles of high m/z range permethylated N‐linked glycans derived from CHO‐K1, CHO‐S, CHO‐K1_mAb_, and CHO‐S_mAb_ cells highlight changes in the abundance of complex N‐glycans (m/z 2500–8000). All molecular ions represent the singly charged and sodiated form [M + Na]^+^. Data were obtained from the 50% acetonitrile (MeCN) fraction from a C_18_ Sep‐Pak. Structures shown with brackets have not had their antennal location unequivocally defined. Some N‐glycan structures are not annotated in these spectra in the interest of clarity—fully annotated spectra for these samples are shown in Figures S1–S4. The *y*‐axis on the right‐hand side shows the relative intensity of the largest signal compared with the largest signal in the m/z 1500–2500 range from the same spectrum (shown in Figure 1).

**Figure 3:**
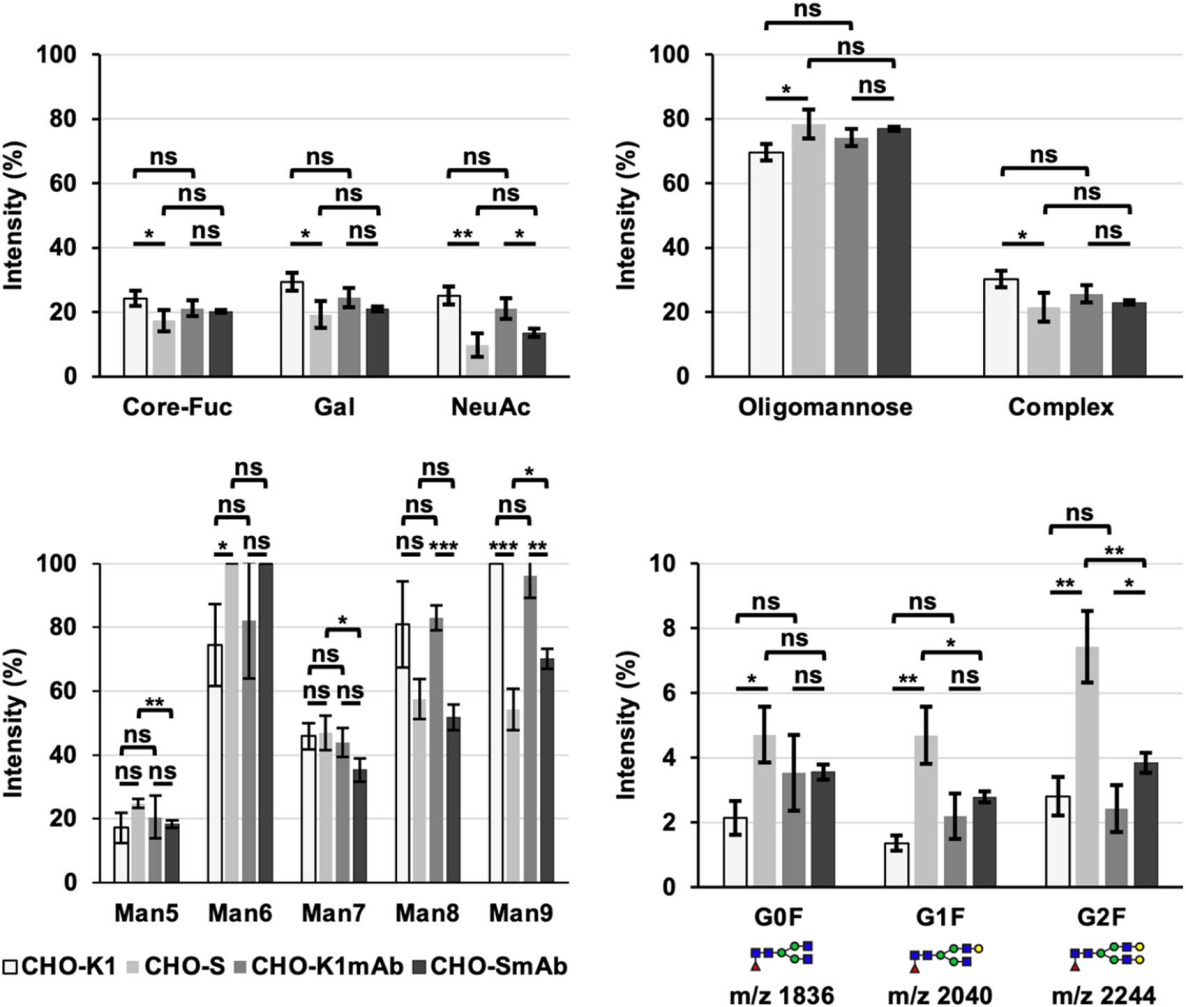
Relative N‐glycan composition of CHO‐K1, CHO‐S, CHO‐K1_mAb_, and CHO‐S_mAb_ cell lines. This data are representative of the 50% MeCN fraction and the mean of three biological replicates. Error bars represent ± 1 standard deviation (SD). The results of two‐tailed unpaired *t*‐tests are shown above the columns (*n* = 3/group, nonsignificant (ns) = *p* > 0.05, *p < 0.05, **p < 0.01, ***p < 0.001). The bottom left panel shows data comparing abundances of oligomannose N‐glycans containing 5–9 mannose residues (m/z: Man_5_ = 1579, Man_6_ = 1783, Man_7_ = 1988, Man_8_ = 2192, Man_9_ = 2396). Data in the bottom right panel compare the abundances of complex, bi‐antennary, and core‐fucosylated N‐glycans containing 0–2 galactose residues (m/z: G0F = 1836, G1F = 2040, G2F = 2244).

The low m/z range of the N‐glycan spectra of CHO‐K1, CHO‐S, CHO‐K1_mAb_, and CHO‐S_mAb_ cells shows prominent signals corresponding oligomannose structures Man_5_GlcNAc_2_ to Man_9_GlcNAc_2_ (m/z 1579, 1783, 1988, 2192, and 2396), with a range of 70%–78% of summed monoisotopic peak intensities (see Figures 1 and 3, Table 2). Additionally, the N‐glycomes of all the cell lines encompass a diverse array of complex N‐glycans, both with and without core fucose, featuring bi‐, tri‐, or tetra‐antennary branching (see Figures 2 and 3). These structures also exhibit variable‐length antennal LacNAc extensions, with up to four terminal sialic acids residues on tetra‐antennary N‐glycans. Although the majority of sialic acids were NeuAc, some minor peaks suggest the presence of *N*‐glycolylneuraminic acid (NeuGc) as well (e.g., m/z 2996, 3084—see Figures S1–S4 for full annotation). While expression of the enzyme CMP‐*N*‐acetylneuraminic acid hydroxylase, responsible for NeuGc production, has previously been shown to be virtually absent in CHO‐K1 cells, it is thought that some residual activity may be responsible for the presence of NeuGc‐sialylated structures (North et al. 2010; Xu et al. 2011). Fortunately, NeuGc levels in CHO cells and the biotherapeutics produced in these hosts are believed to be negligible, thus avoiding the potentially harmful immunogenic effects of this epitope in humans (Ghaderi et al. 2010, 2012; Tangvoranuntakul et al. 2003). MALDI‐TOF‐TOF MS/MS analyses were used to verify the presence of variably branched structures and polylactosamine extended antennae (see Figures S5 and S6). Additionally, MS/MS compositional analyses confirmed that only core‐fucose was present in CHO cells (North et al. 2010). More broadly, the MS data obtained in this study reflect a similar range of N‐glycan structures to those previously identified in Pro^−^5 CHO cells (North et al. 2010). However, upon closer inspection, the data obtained in this study reveal distinct patterns in the size and antennal branching of N‐glycans. For instance, the sialic acid content of CHO‐K1 and CHO‐S cells is significantly higher than in CHO Pro^−^5 cells. This can be readily observed from the fact that sialylated structures have the highest relative intensities among the complex N‐glycans in CHO‐K1 and CHO‐S cells, whereas in CHO Pro^−^5 cells, asialylated structures were more abundant than the sialylated counterparts (North et al. 2010). These differences can most likely be explained by the impact of genetic divergence and antibody production/secretion on the glycosylation machinery of CHO‐K1 and CHO‐S cells. The inherent genetic variations between CHO cell lines fundamentally influence their posttranslational modification capabilities, as each cell line possesses distinct genetic backgrounds that affect protein processing pathways (Reinhart et al. 2018). Multiomics analyses have demonstrated that physiological variations among CHO cell lines cultured under identical conditions stem from underlying genetic deficits that impact cellular processes, including glycosylation machinery (Lakshmanan et al. 2019). These genetic differences result in varied expression patterns of glycosylation enzymes, ultimately affecting the N‐glycan profiles produced by different CHO cell lines (Lewis et al. 2013). Additionally, it has been suggested that the relative abundance of glycosylation machinery to cellular secretory capacity may be a critical determinant of glycosylation efficiency, linking the genetic expression profile to higher‐level cellular dynamics (Jimenez del Val et al. 2016). Advanced network modeling approaches, such as CHOGlycoNET, have further elucidated how the spatial organization and enzymatic distribution within the Golgi apparatus contribute to these cell line‐specific glycosylation patterns, providing mechanistic insights into the relationship between genetic variation and glycan heterogeneity (Kotidis et al. 2023).

**Table 2:** N‐Glycan composition of CHO‐K1, CHO‐S, CHO‐K1_mAb_, and CHO‐S_mAb_ cell lines.

N‐Glycan type	CHO‐K1		CHO‐K1_mAb_		CHO‐S		CHO‐S_mAb_	
	%	S.D.	%	S.D.	%	S.D.	%	S.D.
Oligomannose	70	3	74	3	78	4	77	1
Complex	30	3	26	3	22	4	23	1
Core‐fucosylated	24	2	21	2	17	3	20	0.3
Galactosylated	29	3	24	3	19	4	21	1
NeuAc sialylated	25	3	21	3	10	4	14	1

*Note:* The data are derived from the 50% acetonitrile (MeCN) fraction and represent the mean of three biological replicates.

### 3.2 Differential Oligomannose N‐Glycan Processing in CHO‐K1 and CHO‐S Cells

The CHO‐K1 and CHO‐S cell lines show distinct patterns of oligomannose N‐glycan abundances, which are reflected in the respective antibody‐producing daughter cell lines (see Figure 1). In the MALDI‐TOF MS spectra of CHO‐K1 and CHO‐K1_mAb_ cells, we observe the following pattern of growing relative abundances: Man_5_ < Man_7_ < Man_6_ < Man_8_ < Man_9_ (when abbreviated as Man*_x_*, the presence of a GlcNAc_2_ chitobiose core is implied). On the other hand, a different pattern emerges in CHO‐S and CHO‐S_mAb_ cells, where the MS spectra show the abundances of oligomannose N‐glycan signals as: Man_5_ < Man_7_ < Man_8_ < Man_9_ < Man_6_. The lower abundance of Man_8_ and Man_9_ structures in CHO‐K1 and CHO‐K1_mAb_ cells relative to the CHO‐S and CHO‐S_mAb_ cells could suggest increased activity or expression of ER and Golgi‐resident mannosidases in CHO‐S cells. An increased residence time of glycoproteins in mannosidase‐containing ER and Golgi cisternae might also explain these results. A small difference in oligomannose N‐glycan patterns is also present in the two CHO‐S cell lines, where a higher abundance of Man_5_ and Man_7_, and a lower abundance of Man_9_ in the parental cell line suggest increased mannosidase processing relative to the CHO‐S_mAb_ cells.

### 3.3 Complex N‐Glycan Patterns Diverge in CHO‐K1 Versus CHO‐S Cells

Another key difference between CHO‐K1 and CHO‐S cells and the antibody‐producing daughter cell lines is the complexity and abundance of larger structures at high mass ranges (see Figure 2). The largest N‐glycans are observed in the MALDI‐TOF MS profiles of CHO‐K1 and CHO‐K1_mAb_ cells, where N‐glycans with up to 12 LacNAc moieties (m/z 7820) are clearly resolved. This is a significantly higher mass range than what is resolved in MS spectra for CHO‐S cells, which contain signals corresponding to N‐glycans with up to 9 LacNAc moieties (m/z 5388, see Figure S2 for full annotation). Interestingly, the CHO‐S_mAb_ cells appear capable of generating larger structures at higher mass ranges than the parental CHO‐S cells, where the largest N‐glycan signals resolved are m/z 7633 (see Figure S4 for full annotation) and m/z 5485, respectively. While the mass range for CHO‐S_mAb_ cells is slightly smaller than in the CHO‐K1 and CHO‐K1_mAb_ cell glycoprofiles, the largest N‐glycans contain up to 14 LacNAc moieties (m/z 7633). Overall, an analysis of normalized intensities confirms that CHO‐K1 and CHO‐K1_mAb_ cells produce more complex N‐glycans (26%–30%) than CHO‐S and CHO‐S_mAb_ cells (22%–23%) (see Figure 3), however the only difference found to be statistically significant was between CHO‐K1 (30%) and CHO‐S (22%) cells (*p < 0.05). The greater abundance of complex structures is also apparent from the relative intensity of the largest signal in the m/z 2500–8000 range compared to the m/z 1500–2500 range, which is ~3× higher in the CHO‐K1 and CHO‐K1_mAb_ cell lines (36% and 30%) than in the CHO‐S and CHO‐S_mAb_ cell lines (11% and 10%) (see blue *y*‐axis in Figure 2). These results suggest that the increased mannosidase trimming in CHO‐S and CHO‐S_mAb_ cells does not necessarily translate into increased downstream processing by glycosyltransferases. On the other hand, the larger mass range observed for CHO‐S_mAb_ cells relative to CHO‐S cells is not reflected in the overall increase in the abundance of complex structures (23% and 22%, respectively), which was slight and not statistically significant. Interestingly, CHO‐K1 cells appear to have a higher abundance of complex glycans than CHO‐K1_mAb_ cells, whereas the inverse pattern was observed for CHO‐S and CHO‐S_mAb_ cells. However, neither pairwise comparison was statistically significant.

### 3.4 A Comparison of Fucosylation, Galactosylation, and Sialylation Levels

The higher abundance of complex N‐glycans in the glycoprofiles of CHO‐K1 and CHO‐K1_mAb_ cells relative to CHO‐S and CHO‐S_mAb_ cells is also reflected in a higher level of core‐fucosylated, galactosylated, and sialylated structures (see Figure 3). With regards to fucosylation, CHO‐K1 cells have more core‐fucosylated N‐glycans than CHO‐S cells (24% vs. 17%, *p < 0.05). Similarly, CHO‐K1 cells have more galactosylated N‐glycans than CHO‐S cells overall (29% vs. 19%, *p < 0.05). The other pairwise comparisons for core‐fucosylation and galactosylation were not statistically significant. An analysis of the core‐fucosylated, biantennary agalactosylated, mono‐galactosylated, and di‐galactosylated N‐glycans (G0F, G1F, and G2F, respectively), reveals that all three structures are present in greater abundance in CHO‐S cells compared to the other cell lines (see Figure 3). The smaller mass range and the lower abundance of larger complex and highly elongated N‐glycans observed in CHO‐S cells are likely to explain the increased relative abundance of these smaller N‐glycans. Similarly to the differences in core‐fucosylation and galactosylation, NeuAc sialylated structures are significantly more abundant in CHO‐K1 cells (25%) than in CHO‐S cells (10%) (**p < 0.01). Additionally, the CHO‐K1_mAb_ cells also show a statistically significant increase in sialylation compared to CHO‐S_mAb_ cells (21% vs. 14%, *p < 0.05). A good example of this increased sialylation can be observed when comparing the ratios of the m/z 2605 and 2966 signals, which correspond to mono‐, and di‐sialylated, core‐fucosylated biantennary complex N‐glycans, respectively (see Figure 2). Indeed, in CHO‐K1 and CHO‐K1_mAb_ cells, the peak for the disialylated N‐glycan at m/z 2966 is roughly 3× larger than the m/z 2605 peak, whereas in CHO‐S and CHO‐S_mAb_ cells, the m/z 2966 signal is actually the smaller of the two.

The contrast between the antibody‐producing cell lines is less pronounced than the differences between their parental CHO‐K1 and CHO‐S cell lines. This observation is linked to another pattern: antibody production has inverse effects on the levels of complex N‐glycans in these cell lines, although as mentioned previously, only differences in sialylation levels were found to be statistically significant. In fact, core‐fucosylation, galactosylation, and sialylation levels are lower in CHO‐K1_mAb_ cells than CHO‐K1 cells, whereas the opposite applies to CHO‐S_mAb_ and CHO‐S cells. It is difficult to explain exactly why these opposing trends are observed and further investigation is warranted. One hypothesis is that if antibody production does influence N‐glycosylation in CHO cells, it is likely to be linked to the specific mAb productivity of the cell, which was not investigated in this study. We envisage that specific mAb productivity is likely to affect glycosylation enzyme saturation and glycoprotein residence time in various Golgi cisternae along the secretory pathway, which could affect N‐glycan processing in host cell glycoproteins (Jimenez del Val et al. 2016). Additionally, the consumption of cellular resources for mAb production might affect the expression of glycosyltransferases, glycosidases, sugar transporters, and upstream metabolic pathways linked to glycosylation. For example, when the specific productivity of various biopharmaceuticals was increased in CHO cells via temperature reduction or sodium butyrate supplementation, variable effects on glycosylation were reported (Ahn et al. 2008; Fox et al. 2004; Hendrick et al. 2001; Kantardjieff et al. 2010; Spearman et al. 2005; Sung et al. 2004; Trummer et al. 2006). These results were generally considered a consequence of pleiotropic effects linked to cell cycle arrest. Conversely, some articles suggest that increased specific productivity is linked with reduced glycan processing (Fan et al. 2015; Santell et al. 1999; Trummer et al. 2006). One group in particular concluded that this was likely to be a result of reduced availability of glycosylation machinery relative to the secretory capacity of the cell (Fan et al. 2015; Jimenez del Val et al. 2016). The underlying mechanistic details of how mAb production might influence cellular N‐glycosylation and biopharmaceutical glycosylation are poorly understood. Further studies will be necessary for understanding whether effects are cell line‐specific and how glycosylation machinery availability, Golgi apparatus size, and retention time might affect glycosylation capabilities.

### 3.5 Endo‐β‐Galactosidase Digests Reveal Further Differences Between CHO‐K1 and CHO‐S Cells and the Impact of Antibody Production on N‐Glycosylation

**Figure 4:**
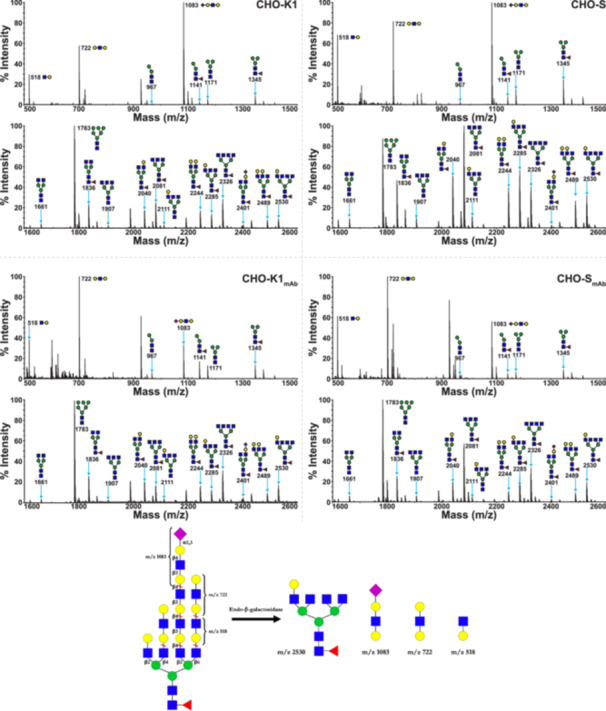
MALDI‐TOF MS profiles of permethylated N‐linked glycans derived from CHO‐K1, CHO‐S, CHO‐K1_mAb_, and CHO‐S_mAb_ cells after endo‐β‐galactosidase digestion of released N‐glycans. All molecular ions represent the singly charged and sodiated form [M + Na]^+^. Data in the m/z 500–1500 range were obtained from the 35% MeCN fraction from a C_18_ Sep‐Pak. Data in the m/z 1600–2600 range were obtained from the 50% MeCN fraction. Below the MALDI‐TOF MS spectra is a diagram showing an example of the expected N‐glycan and released antennal structures resulting from endo‐β‐galactosidase digestion of a complex N‐glycan.

An additional level of structural characterization of the N‐glycome in CHO‐K1, CHO‐S, CHO‐K1_mAb_, and CHO‐S_mAb_ cells was carried out using endo‐β‐galactosidase digests to better determine differences in nonreducing end terminal motifs, and branching patterns. Endo‐β‐galactosidase hydrolyzes internal β1‐4 linkages in linear LacNAc repeats ([GlcNAc β1‐3Galβ1‐4]_*n*_) and the postdigestion MALDI‐TOF MS spectra are presented in Figure 4. The enzyme treatment generates signals corresponding to the structures of trimmed antennal motifs (m/z 518—GlcNAcGal, m/z 722—GalGlcNAcGal, and m/z 1083—NeuAcGalGlcNAcGal), which provide an additional data set for comparing the relative abundances of galactosylated and sialylated polylacNAc antennae. Additionally, endo‐β‐galactosidase digests also produce bi‐, tri‐, and tetra‐antennary trimmed core N‐glycans with signals at m/z 1836, 2081, 2285, 2326, and 2530 that can help the comparison of branching levels of polylacNAc containing glycans across the cell lines. The results show that these CHO cell lines present polylactosamine extensions on a mixture of bi‐, tri‐, and tetra‐antennary N‐glycans, similarly to previous findings (North et al. 2010).

**Figure 5:**
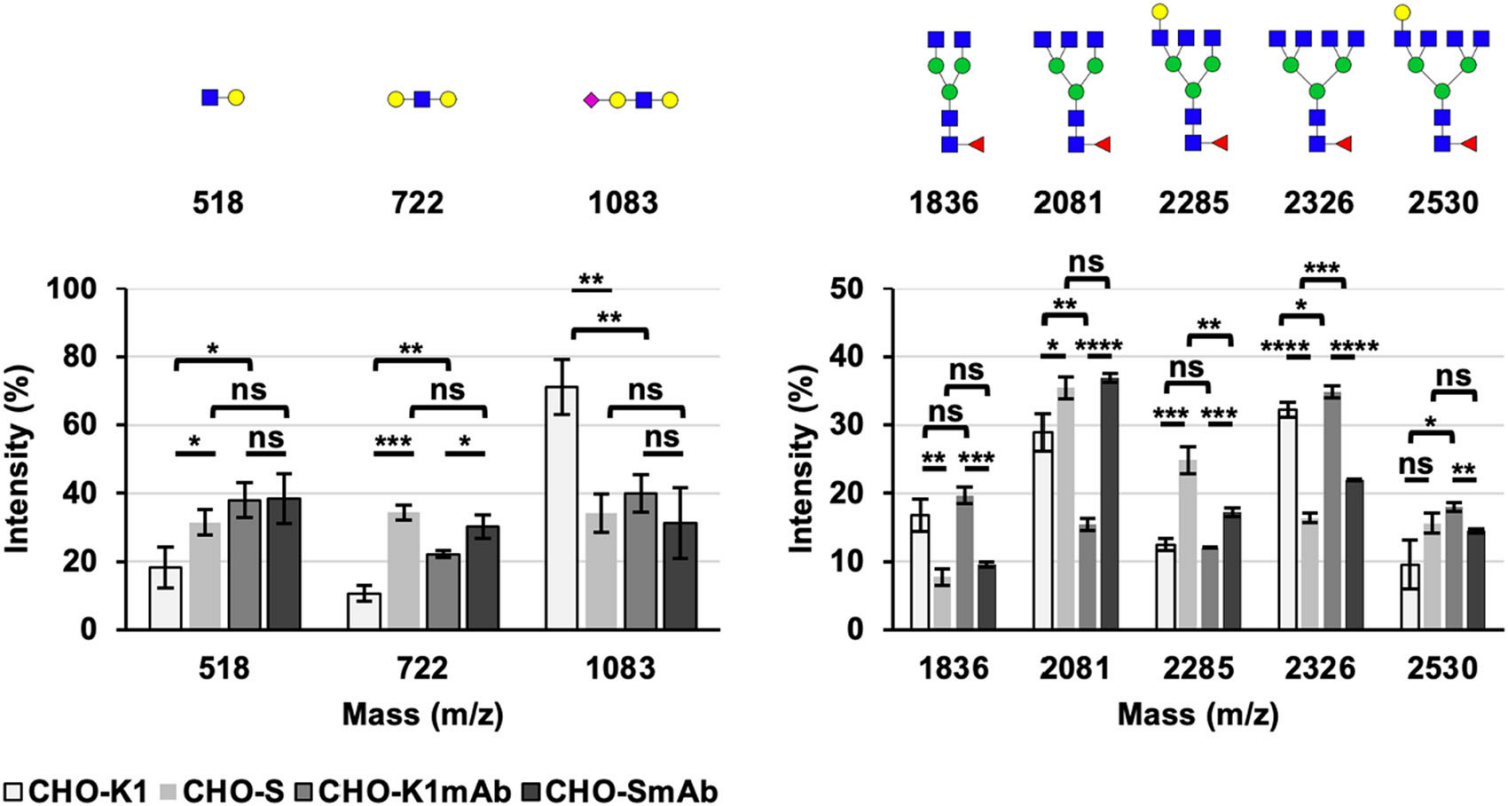
Graphical detail of terminal motif distribution and antennarity of N‐glycans, after endo‐β‐galactosidase digest, from CHO‐K1 and CHO‐S cell lysates compared with antibody‐producing CHO‐K1_mAb_ and CHO‐S_mAb_ cells. The left‐hand graph represents N‐glycan profiles obtained from 35% MeCN fractions from a C_18_ Sep‐Pak, while the right‐hand graph refers to the 50% MeCN profiles. Terminal structures with m/z 518, 722, and 1083 do not elute well in the 50% MeCN fraction, which is why these data are not shown. The structures assigned to the various molecular ions are shown. The relative intensities are the mean of three biological replicates and error bars represent ± 1 SD. The results of two‐tailed unpaired *t*‐tests are shown above the columns (*n* = 3/group, nonsignificant (ns) = *p* > 0.05, *p < 0.05, **p < 0.01, ***p < 0.001, ****p < 0.0001).

The semi‐quantitative analysis of N‐glycans from digested samples revealed that LacNAc extensions on tri‐antennary structures were significantly more abundant in CHO‐S and CHO‐S_mAb_ cells (e.g., m/z 2081) compared to CHO‐K1 and CHO‐K1_mAb_ cells, whilst polylactosamine repeats were considerably more abundant in bi‐ and tetra‐antennary N‐glycans (e.g., m/z 1836, 2326) in the latter pair (see Figure 5). This contrasts with previous data from Pro^−^5 CHO cells, where polylactosamine extensions were found primarily on biantennary N‐glycans (North et al. 2010). Interestingly, both mAb‐producing cell lines show a greater abundance of tetra‐antennary N‐glycans and a lower abundance of tri‐antennary structures than in parental cell lines. More specifically, CHO‐K1_mAb_ cells show a greater abundance of the tetra‐antennary N‐glycan signal at m/z 2530 (*p < 0.05) and a lower abundance of the tri‐antennary N‐glycan at m/z 2081 (**p < 0.01) compared with CHO‐K1 cells. Similarly, the levels of tetra‐antennary structures were higher in CHO‐S_mAb_ cells than the parental CHO‐S cells (m/z 2326, ***p < 0.001), whereas tri‐antennary N‐glycans were less abundant (m/z 2285, **p < 0.01). Overall, these findings indicate a correlation between antibody production and increased branching in N‐glycans in both CHO‐K1 and CHO‐S cells.

In line with data from the undigested samples, the cleaved sialylated terminal motif at m/z 1083 is more abundant (~2×, **p < 0.01) in CHO‐K1 cells than in CHO‐S cells (see Figure 5), confirming the greater sialylation levels in CHO‐K1 cells. A lower abundance of the terminally galactosylated motif at m/z 722 in CHO‐K1 cells relative to CHO‐S cells (***p < 0.001) is further evidence of greater sialyltransferase activity in the K1 lineage. The same difference is observed between the antibody‐producing cell lines, although it is less pronounced (*p < 0.05). Sialylation levels based on the m/z 1083 terminal motif were also found to be lower in CHO‐K1_mAb_ cells relative to the progenitor CHO‐K1 cells (**p < 0.01), which reflects findings from the undigested samples. The lower abundance of uncapped terminal motifs at m/z 518 and 722 in CHO‐K1 cells compared with the antibody‐producing daughter cell line (*p < 0.05 and **p < 0.01, respectively) lends support to the hypothesis that antibody production is correlated with decreased sialylation in CHO‐K1 cells.

### 3.6 A Comparison of Glycosylation Capabilities in CHO‐K1 and CHO‐S Cells as Seen Through Recombinantly Produced IgG1‐Fc Constructs

**Figure 6:**
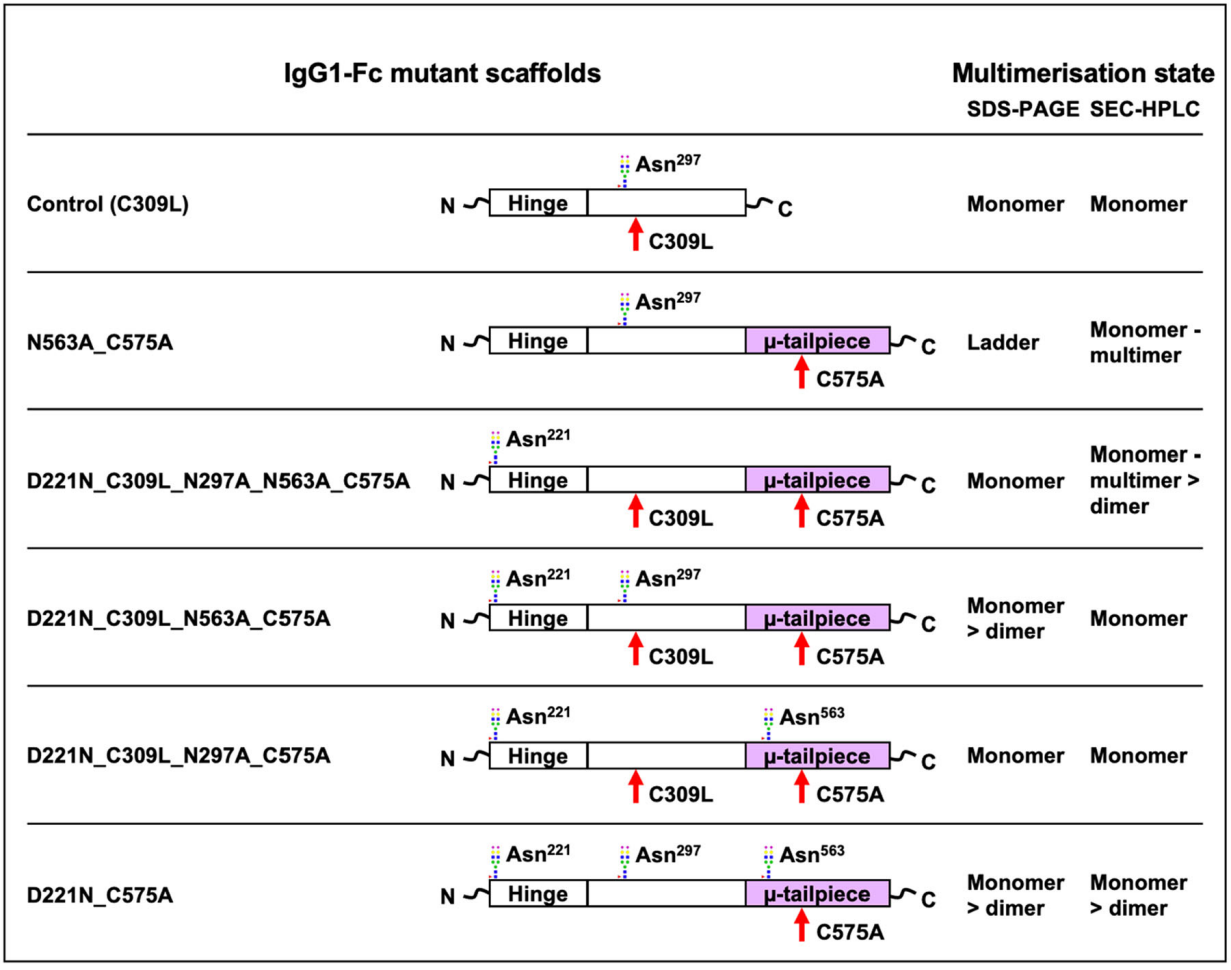
Schematic showing the various IgG1‐Fc glycosylation mutants produced in CHO‐K1 and CHO‐S cells. Cys^575^ is mutated to alanine to create the C575A panel of mutants. Additionally, Cys^309^ and Leucine^310^ are changed to leucine and histidine, respectively, as found in the native IgG1‐Fc sequence to create the C309L mutants. N‐glycans mark the approximate location of the hinge Asn^221^, the C_H_2 Asn^297^, and the IgM (μ) tailpiece Asn^563^ glycosylation sites. The multimeric state of the IgG1‐Fc mutants is shown for IgG1‐Fc mutants produced in CHO‐K1 cells (Blundell et al. 2019).

In addition to comparing the cellular N‐glycosylation capabilities of CHO‐K1 and CHO‐S cells, we used MALDI‐TOF MS to compare N‐glycomic profiles from a series of IgG1‐Fc constructs produced in CHO‐S cells to previously obtained data for the same constructs in CHO‐K1 cells (Blundell et al. 2019, 2020). Protein backbone mutations are an established glycoengineering strategy, which can be used to add novel glycosylation sites, modulate accessibility to glycosylation enzymes, and enable novel glycan functionalities via multimerization. The IgG1‐Fc constructs in question were selected from a panel with variable multimerization abilities that depend on the insertion of an IgM µ‐tailpiece at the C‐terminus, the mutation of cysteine residues (C309L and C575A), and the insertion of novel N‐glycosylation sites at Asn^221^ and Asn^563^ (see Figure 6) (Blundell et al. 2017, 2019, 2020; Czajkowsky et al. 2015; Mekhaiel et al. 2011). A monomeric IgG‐Fc with only the classical Asn^297^ site and a C309L mutation was used as a reference. The remaining five constructs contained a C‐terminal µ‐tailpiece and a mixture of the following mutations C309L, C575A, D221N, N297A, and N563A, which modulate multimerization and the number of glycosylation sites. Interestingly, the Asn^221^ site was previously shown to contain hypersialylated N‐glycans, which is unusual in IgG‐Fc N‐glycans normally found at Asn^297^ (Blundell et al. 2017). These hypersialylated N‐glycans enabled strong binding to sialic acid‐dependent receptors and were also found to inhibit influenza A and B‐induced hemagglutination of human erythrocytes when produced in CHO‐K1 cells (Blundell et al. 2017, 2019, 2020). However, when produced in HEK‐293 cells, which express both α2,3‐ and α2,6‐linked NeuAc, the constructs were not able to inhibit influenza B‐induced hemagglutination, which highlights the importance of cell line‐specific glycosylation capabilities (Blundell et al. 2020). While human cells can generate N‐glycan structures not seen in CHO cells (e.g., antennal fucosylation or α2,6‐linked NeuAc), the differences between CHO‐K1 and CHO‐S cells are more subtle. The results that follow will illustrate the importance of understanding the glycosylation capabilities of different CHO cell lineages in manufacturing biopharmaceuticals.

**Figure 7:**
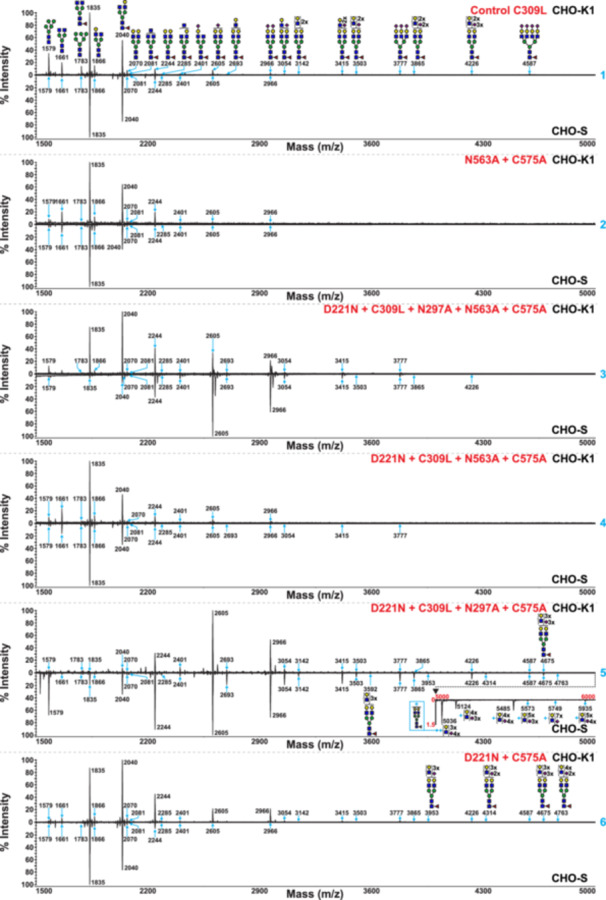
MALDI‐TOF mass spectrometry profiles of permethylated N‐glycans from human IgG1‐Fc constructs produced in CHO‐S and CHO‐K1 cells. All molecular ions represent the singly charged and sodiated form [M + Na]^+^. Structures shown with brackets have not had their antennal location unequivocally defined.

**Figure 8:**
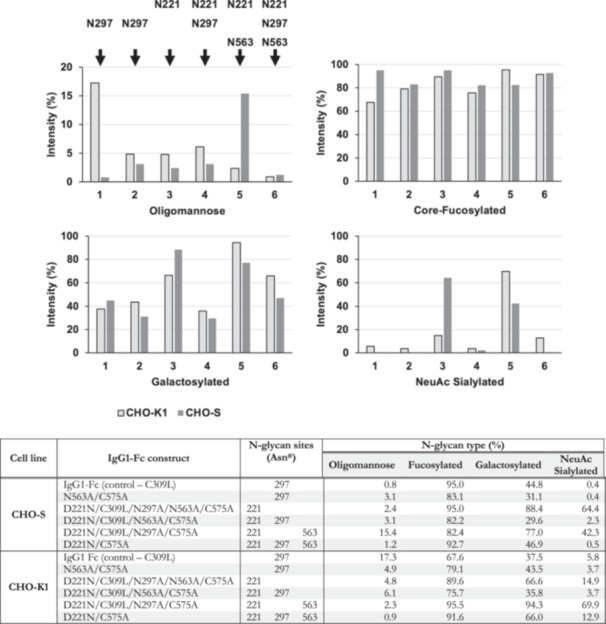
N‐glycan compositions of human IgG1‐Fc constructs produced in CHO‐K1 and CHO‐S cells. The different IgG‐Fc constructs are listed as numbers according to the order in Figure 6. 1 = Control (C309L); 2 = N563A_C575A; 3 = D221N_C309L_N297A_N563A_C575A; 4 = D221N_C309L_N563A_C575A; 5 = D221N_C309L_N297A_C575A; 6 = D221N_C575A. The N‐glycosylation sites present in each construct are reiterated above the top left panel and in the table.

The N‐glycomic MALDI‐TOF MS spectra of IgG1‐Fc constructs produced in CHO‐S cells were obtained and compared to previously reported data from CHO‐K1 cells (see Figure 7). As expected from the constructs produced in CHO‐K1 cells, CHO‐S cell constructs containing solely the Asn^297^ N‐glycosylation site exhibit less processed N‐glycans than constructs containing Asn^221^ and Asn^563^. A semi‐quantitative analysis illustrates this further, where constructs containing only Asn^221^ and Asn^563^ have a higher abundance of galactosylation (66.6%–94.3%) and sialylation (14.9%–69.9%) than Asn^297^‐containing constructs (Gal 31.1%–44.8%, NeuAc 0.4%–5.8%) (see Figure 8). While the sialylation levels of Asn^297^‐only constructs from CHO‐K1 cells are low (3.7%–5.8%), sialylation was found to be virtually absent in the same IgG1‐Fc constructs from CHO‐S cells (0.4%). This can also be observed from the smaller N‐glycan mass range, which extends up to m/z 2966 in CHO‐S cells compared to m/z 4587 in CHO‐K1 cells for the control C309L construct. The abundance of galactosylated N‐glycans was also found to be higher in the Asn^297^ only construct N563A/C575A when produced in CHO‐K1 cells (43.5% vs. 31.1%). Interestingly, in the Asn^297^‐only control Fc galactosylation levels were lower in CHO‐K1 cells (37.5%) compared to CHO‐S cells (44.8%). However, when oligomannose N‐glycans are excluded from the semi‐quantitative analysis, galactosylation levels are largely similar (45.2%–45.3%). With respect to the abundance of core‐fucosylated N‐glycans, no clear pattern was discerned, and there is considerable variability between Fc constructs (67.7%–95.5%). Additionally, the differences in core‐fucosylation between CHO‐K1 and CHO‐S cells are relatively small. It is noted that while minor NeuGc‐containing structures were identified in low abundances, particularly in hypersialylated constructs, these were not considered for semi‐quantitation.

Overall, four of the six constructs produced in CHO‐K1 cells have a higher abundance of galactosylated N‐glycans, and five have a higher abundance of sialylated N‐glycans compared to constructs produced in CHO‐S cells, which partially aligns with the findings from the host cell N‐glycome experiments reported above. Another example of this pattern is the construct containing Asn^221^ and Asn^563^ (D221N/C309L/N297A/C575A), where galactosylation and sialylation levels were higher in CHO‐K1 cells (94.3% Gal, 69.9% NeuAc) relative to CHO‐S cells (77.0% Gal, 42.3% NeuAc), despite the higher mass range extending up to m/z 5935 observed in the latter (it is noted that in the previous study, data were not obtained above m/z 5000 for CHO‐K1 cells). However, the Asn^221^‐only construct (D221N/C309L/N297A/N563A/C575A) produced in CHO‐S cells bucks this trend and shows increased galactosylation (88.4% vs. 66.6%) and sialylation (64.4% vs. 14.9%) levels compared to the construct produced in CHO‐K1 cells. Cell line‐specific glycosylation capabilities or natural variation could both explain these opposing patterns.

The oligomannose content of constructs produced in CHO‐K1 cells was generally found to be higher than in CHO‐S cells, except for constructs containing the Asn^563^ site (D221N/C309L/N297A/C575A and D221N/C575A). However, the overall pattern in oligomannose content across the constructs and cell lines is unclear. In fact, high‐mannose N‐glycans are mostly present in low abundances (0.8%–6.1%), yet the control Fc from CHO‐K1 cells (17.3%) and the D221N/C309L/N297A/C575A construct from CHO‐S cells (15.4%) contain unusually high levels of oligomannose structures. We cannot say why such differences in oligomannose processing emerged between the constructs; however, redox‐sensitive cysteine residues have been suggested as a possible influence on glycosylation (Blundell et al. 2020; Capellari et al. 1999).

Hypersialylated IgG1‐Fc constructs produced in CHO‐K1 cells could eventually serve as a basis for novel biopharmaceuticals that target autoimmune diseases via sialic acid receptor binding and treat viral influenza infections via hemagglutinin engagement. In light of the lower overall sialylation levels for constructs produced in CHO‐S cells relative to CHO‐K1 cells, CHO‐S cells might not be a suitable production platform for such hypersialylated therapies.

## 4 Conclusions

The general conclusions of this study are that CHO‐K1 cells have a greater ability to process N‐glycans into larger and more complex structures than CHO‐S cells. This is reflected particularly in the higher abundance of sialylated N‐glycans and the increased level of branching in CHO‐K1 cells. The endo‐β‐galactosidase digests provided further confirmation of these patterns and also revealed that antibody production was associated with increased branching in both CHO‐K1 and CHO‐S cells and reduced sialylation in CHO‐K1 cells. Additionally, the differences in the cellular N‐glycomes were mirrored in a comparison of N‐glycans from a panel of IgG1‐Fc constructs produced in CHO‐K1 and CHO‐S cells. Our results suggest that CHO‐K1 cells may be better suited for the manufacture of biopharmaceuticals where more complex, branched, or sialylated N‐glycans are beneficial. Ultimately, we have shown that decades of genetic divergence in industrial CHO cell lines are likely to have impacted glycosylation capabilities. Looking forward, further studies could elucidate whether natural variation, cell line‐specific glycosylation capabilities or specific productivity are responsible for some of the trends observed in the data. In future, it would also be of interest to determine whether cell line‐specific differences are present in the O‐glycosylation profiles of industrially relevant CHO cells, which could help to inform the choice of cell line for O‐glycosylated biologics. Finally, understanding the glycosylation machinery of CHO cells is essential for the development of predictive in silico models for cellular and biopharmaceutical glycosylation, and will ultimately help improve the effectiveness of glycoengineering strategies and the development of biobetters.

## Author Contributions

Cleo Kontoravdi, Stuart M. Haslam, Anne Dell, and Roberto Donini conceptualized the study and developed the experimental design. Experiments were performed by Roberto Donini, Pat Blundell, and Dongli Lu. Data processing, analysis, and interpretation were performed by Roberto Donini, Pat Blundell, Richard J. Pleass, Dongli Lu, Anne Dell, Cleo Kontoravdi, and Stuart M. Haslam. Roberto Donini prepared all figures and tables. Roberto Donini, Cleo Kontoravdi, and Stuart M. Haslam wrote the first draft of the manuscript. All authors critically reviewed, read, and approved the final manuscript.

## Supporting information

Supp_Figures.

## Data Availability

The data that support the findings of this study are available from the corresponding author upon reasonable request.
