# Supplementary material for: Genetic Divergence and Antibody Expression Influence the N‐Glycomes of CHO‐K1 and CHO‐S Cells
Source: Biotechnol Bioeng. 2025 Aug 16;122(11):3160–75. doi: 10.1002/bit.70045 (PMC12503014; doi:10.1002/bit.70045)
Supplement: Supplementary file 1 — Supp_Figures. [file BIT-122-3160-s001.pdf]

Genetic divergence and antibody expression influence the N-glycomes of CHO-K1 and CHO-S cells.

Roberto Donini[1†], Pat Blundell[2], Richard J. Pleass[2], Dongli Lu[1§], Anne Dell[1], Cleo Kontoravdi[4] & Stuart M. Haslam[1‡]

[1]Department of Life Sciences, Imperial College London, London, United Kingdom
[2]Department of Tropical Disease Biology, Liverpool School of Tropical Medicine, Liverpool, United Kingdom
[4]Department of Chemical Engineering, Imperial College London, London, United Kingdom
†First author
§Current address: Department of Medicine, Cedars Sinai Medical Center, Los Angeles, California, USA
‡Correspondence: s.haslam@imperial.ac.uk

Supplementary Figures

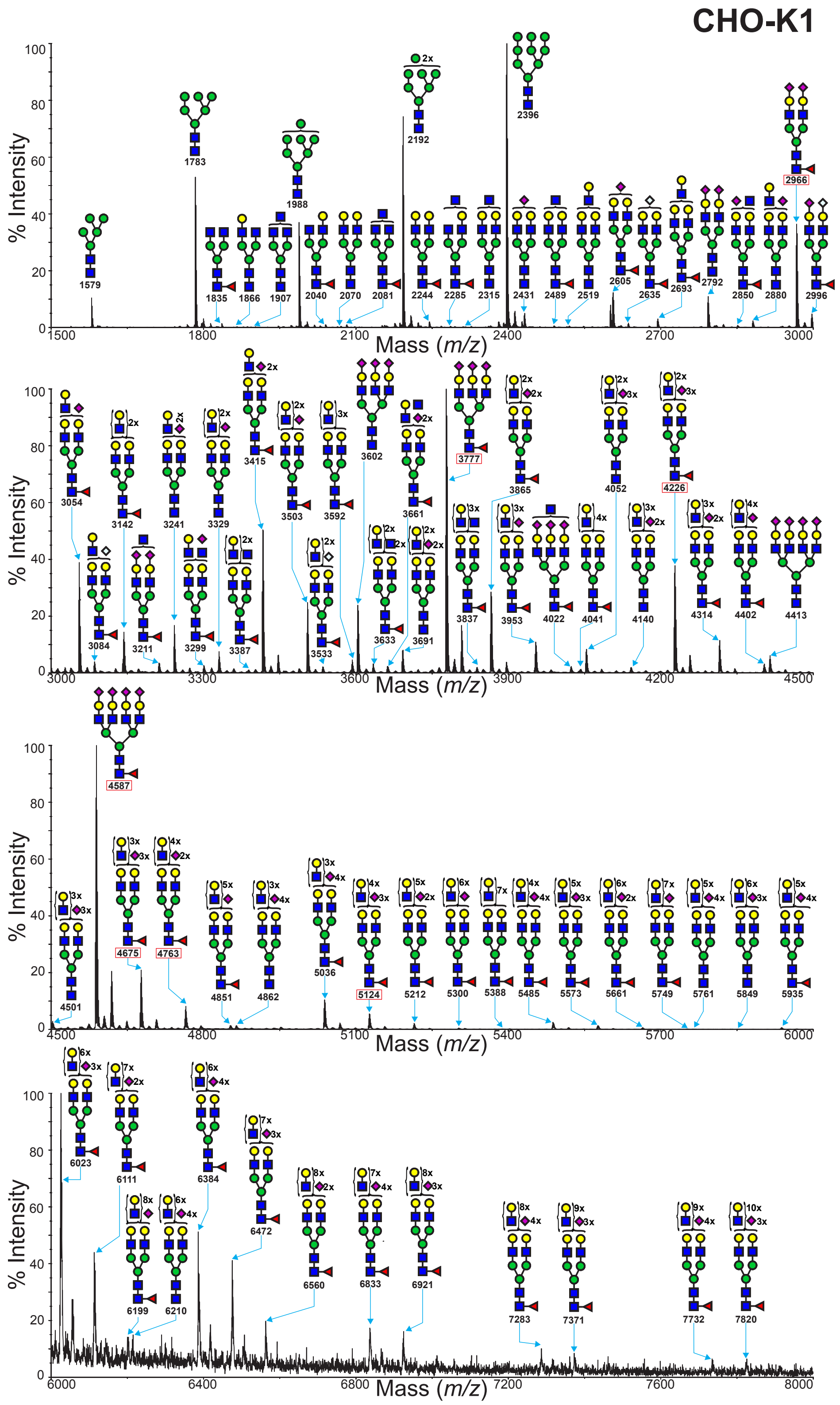

Supplementary Figure 1. MALDI-TOF MS profile of permethylated N-linked glycans derived from CHO-K1 cells.

All molecular ions represent the singly charged and sodiated form $[M + Na]^+$. Data were obtained from the 50% acetonitrile (MeCN) fraction from a C_{18} Sep-Pak. Structures shown with brackets have not had their antennal location unequivocally defined. Signals with m/z values highlighted in red boxes were selected for MS/MS fragmentation analysis (see Supplementary Figures 5-8).

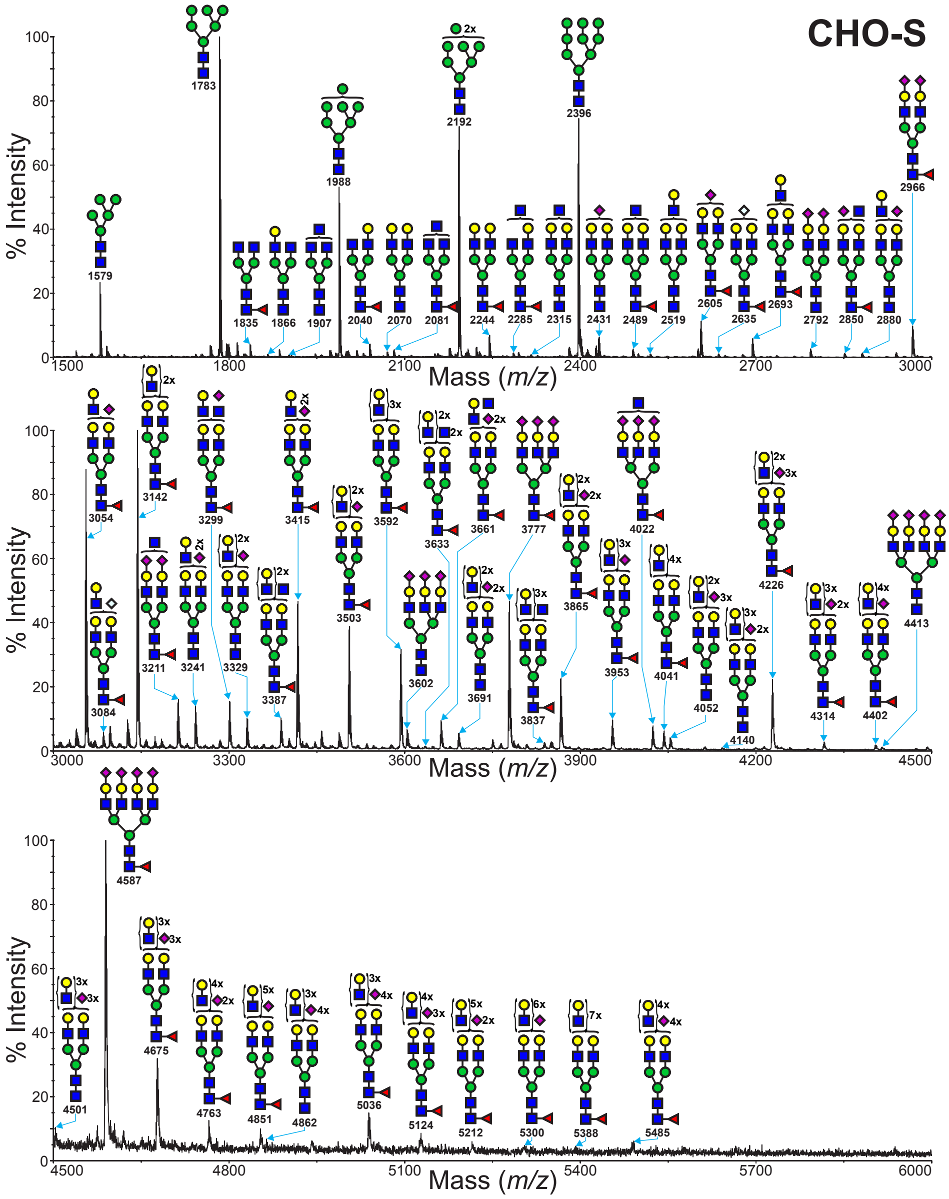

Supplementary Figure 2. MALDI-TOF MS profile of permethylated N-linked glycans derived from CHO-S cells.

All molecular ions represent the singly charged and sodiated form $[M + Na]^{+}$. Data were obtained from the 50% acetonitrile (MeCN) fraction from a C_{18} Sep-Pak. Structures shown with brackets have not had their antennal location unequivocally defined.

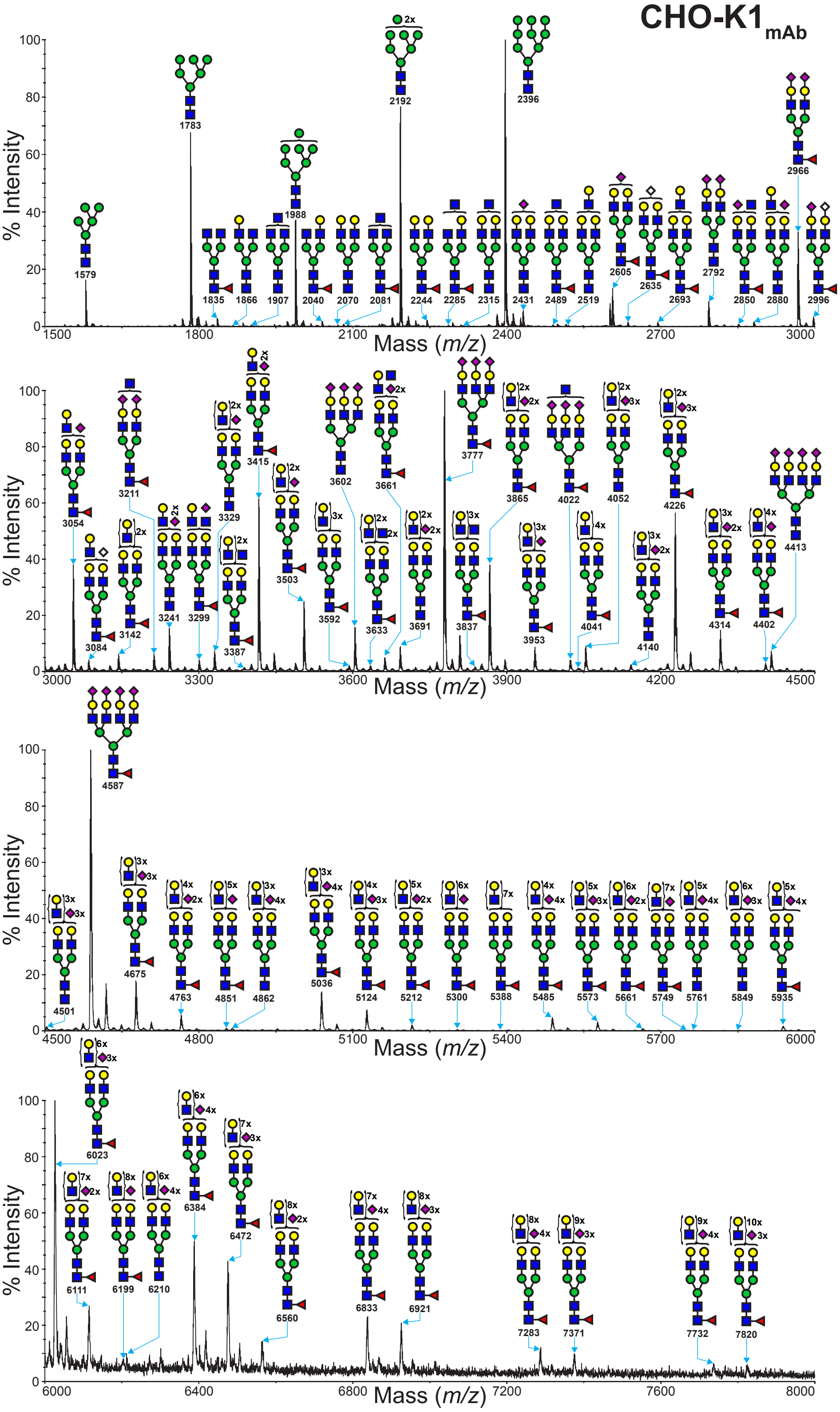

Supplementary Figure 3. MALDI-TOF MS profile of permethylated N-linked glycans derived from CHO-K1$_{mAb}$ cells.

All molecular ions represent the singly charged and sodiated form $[M + Na]^+$. Data were obtained from the 50% acetonitrile (MeCN) fraction from a C_{18} Sep-Pak. Structures shown with brackets have not had their antennal location unequivocally defined.

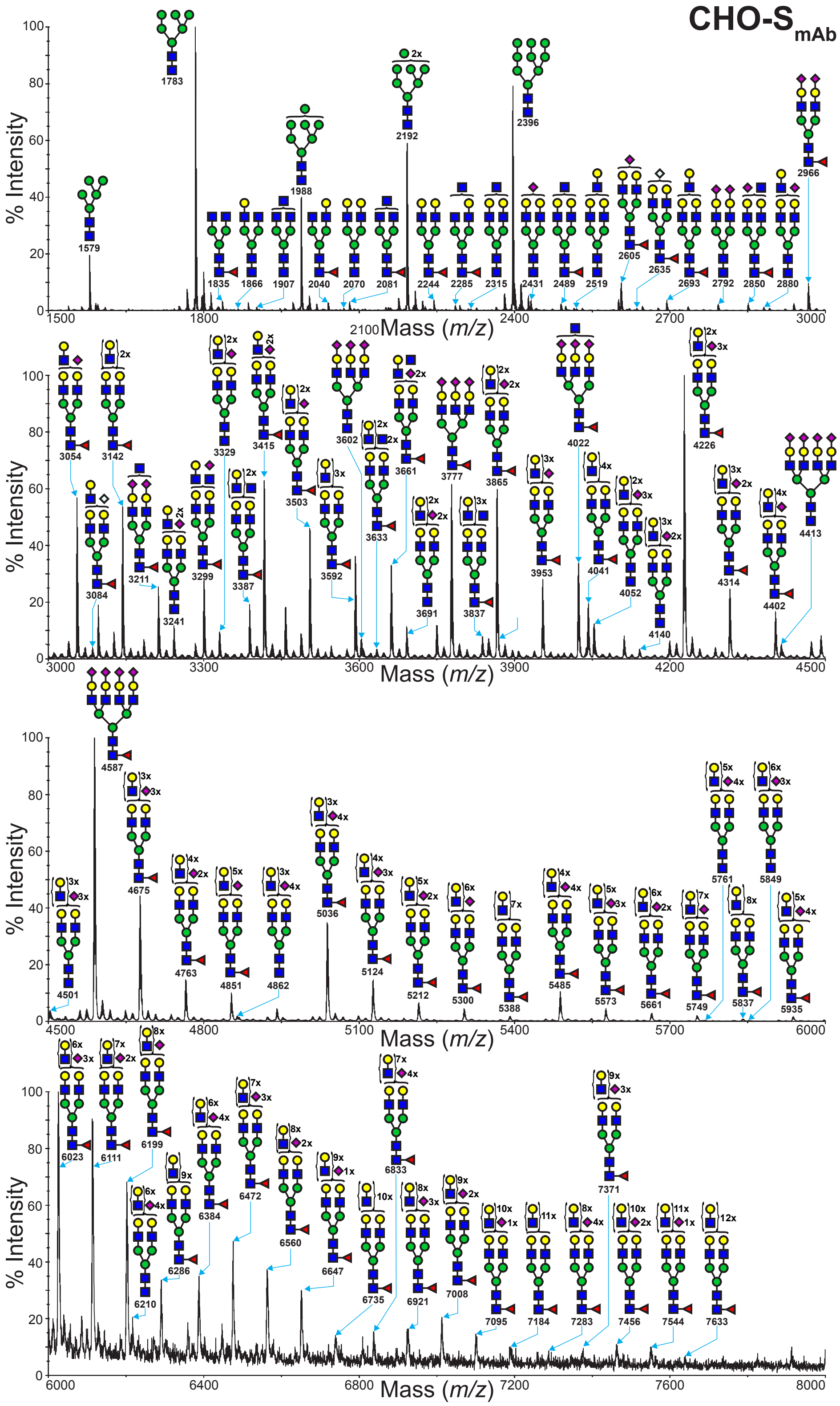

Supplementary Figure 4. MALDI-TOF MS profile of permethylated N-linked glycans derived from CHO-S$_{mAb}$ cells.

All molecular ions represent the singly charged and sodiated form $[M + Na]^+$. Data were obtained from the 50% acetonitrile (MeCN) fraction from a C_{18} Sep-Pak. Structures shown with brackets have not had their antennal location unequivocally defined.

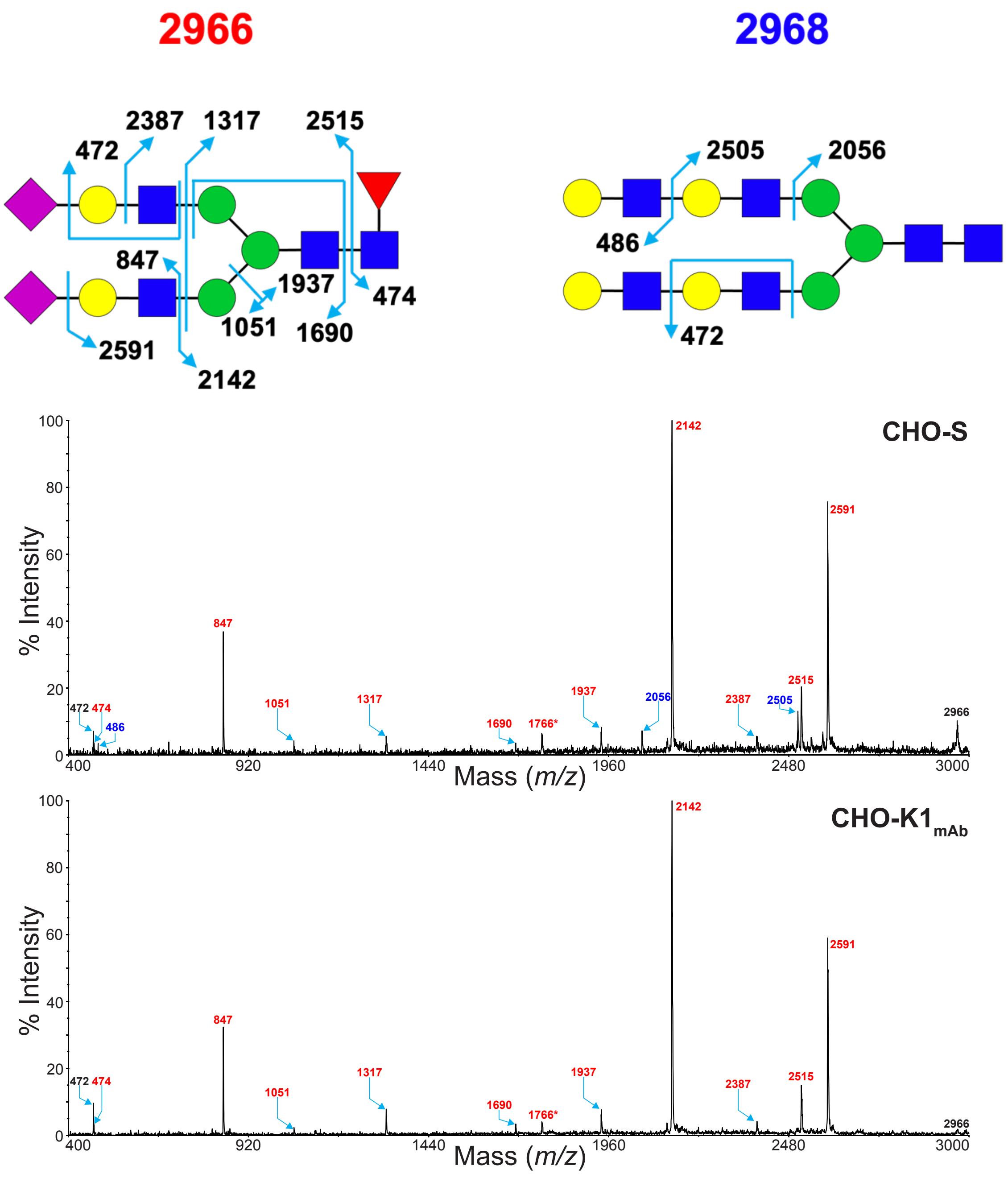

Supplementary Figure 5. MALDI-TOF/TOF MS/MS spectrum of the molecular ion at m/z 2966, derived from the N-glycan spectrum of CHO-S and CHO- K1$_{mAb}$ cells.

There is evidence that the afucosylated structure shown above with a permethylated mass of 2968 is present in the CHO-S spectrum, however in extremely low abundance compared to the fucosylated structure with m/z 2966. There is no evidence of the m/z 2968 N-glycan structure in the CHO-K1$_{mAb}$ MS/MS spectrum. Ion signal m/z values highlighted in red represent fragments exclusively belonging to the fucosylated structure and those in blue belong to the afucosylated structure. Alternative possible antennal arrangements for the N-glycan with m/z 2968 are not shown. The ion highlighted with an asterisk represents a fragment with multiple cleavages and is not illustrated to ensure clear annotation. All ions represent the singly charged and sodiated form.

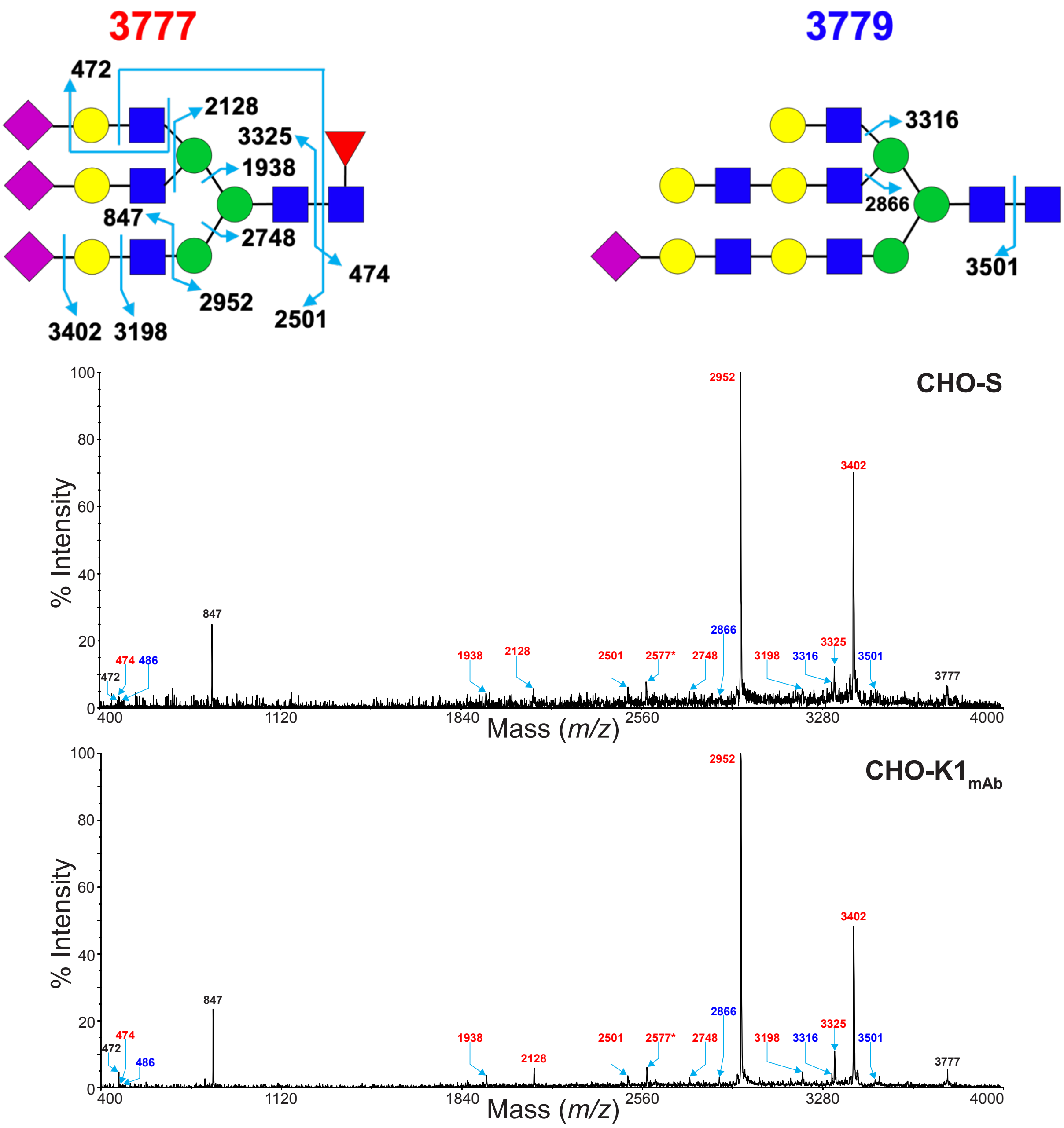

Supplementary Figure 6. MALDI-TOF/TOF MS/MS spectrum of the molecular ion at m/z 3777, derived from the N-glycan spectrum of CHO-S and CHO-K1$_{mAb}$ cells.

The afucosylated structure shown above with a permethylated mass of 3779 may be present, however in extremely low abundance compared to the fucosylated structure with m/z 3777. Ion signal m/z values highlighted in red represent fragments exclusively belonging to the fucosylated structure and those in blue belong to the afucosylated structure. Alternative possible antennal arrangements for the N-glycan with m/z 3779 are not shown. The ion signal at m/z 2577 represents a fragment with multiple cleavages and is not illustrated to ensure clear annotation. All ions represent the singly charged and sodiated form.
